# Promoting Inclusive Learning Environments: Leveraging University Websites for Digital Empowerment in the Post-COVID-19 Era

**DOI:** 10.3390/healthcare12121212

**Published:** 2024-06-18

**Authors:** Walaa Sayed Mohammad, Ali Fahad Aldakhil

**Affiliations:** 1 Department of Physical Therapy, College of Applied Medical Sciences, Majmaah University, Al-Majmaah 11952, Saudi Arabia; 2 Department of Biomechanics, Faculty of Physical Therapy, Cairo University, Giza 11432, Egypt; 3 Special Education Department, College of Education, Majmaah University, Al-Majmaah 11952, Saudi Arabia

**Keywords:** social responsibility, sustainable development, disability empowerment, higher education websites, digital accessibility, COVID-19 pandemic

## Abstract

In an era of global interdependence, universities play a crucial role in promoting social responsibility and sustainable development. The United Nations Convention on the Rights of Persons with Disabilities emphasizes the empowerment of individuals with disabilities, a key aspect of inclusion. The COVID-19 pandemic underscored the necessity of digital platforms to ensure equitable opportunities, particularly for those with disabilities. It highlighted challenges in transitioning to remote operations and stressed the importance of accessible digital tools in maintaining inclusivity during disruptions. This study investigates how university websites promote social responsibility and empower individuals with disabilities in Saudi Arabia, the UK, and the US. It also examines how these websites foster inclusivity, advocate for disability rights, and contribute to societal change. Using a qualitative case study design, the study evaluated university websites focusing on accessibility services, inclusive policies, and academic accommodations. Qualitative content and thematic analyses identified recurring themes and variations. The findings reveal diverse strategies in empowerment initiatives, website maintenance practices, community engagement approaches, the accessibility of support services, and the presentation of success stories. Despite differences among websites, this study underscores their significance in empowering individuals with disabilities. Recommendations aim to guide universities worldwide in enhancing their digital platforms, ensuring inclusivity, especially during challenges like the COVID-19 pandemic.

## 1. Introduction

In an era of remarkable connectivity and international interdependence, the concepts of social responsibility and sustainable development have gained significant prominence across various sectors. Within this context, the empowerment of individuals with disabilities emerges as a cornerstone of inclusivity and progress. According to the World Health Organization (WHO), disability is defined as a dynamic interaction between health conditions and contextual factors, encompassing impairments, activity limitations, and participation restrictions [1]. Within the framework of social responsibility and sustainable improvement, the empowerment of people with disabilities emerges as a cornerstone of inclusivity and development. The United Nations’ Convention on the Rights of Persons with Disabilities (CRPD) underscores the right of individuals with disabilities to full and identical participation in all elements of existence [2]. Empowerment encompasses equitable access to education, employment, healthcare, and social participation, all of which contribute to the realization of an inclusive society [3].

Consequently, as society becomes more aware of diversity and equity, institutions of higher education must promote the rights and empower those with disabilities. A critical component of this effort is the use of university websites as venues for offering information, raising awareness, and showcasing initiatives that make contributions to the overall well-being of disabled people [4]. These websites function as digital gateways to a world of opportunities, information, and resources, with the capacity to inspire change in an increasingly digital society. The World Health Organisation (WHO) defines disability as an altering term impacted by elements such as impairment, activity limits, and societal engagement. It transcends mere physical or cognitive differences, encompassing a complex interaction between personality, skills, and the environmental context in which those persons exist [5]. To this aim, universities are tasked with not only providing accessible physical venues but also fostering a culture that values diversity and upholds the concepts of inclusiveness and empowerment [6].

The backdrop in the direction of which this debate unfolds is defined by the use of a global event that has left an indelible impact on society: the COVID-19 pandemic. Worldwide, the pandemic has challenged individuals, society, and institutions in unparalleled approaches. For individuals with disabilities, the hurdles have regularly been even higher. The fast transition to online education, remote work, and telehealth services has become the new standard, with digital accessibility taking centre stage. The pandemic spotlighted the importance of accessible online content, inclusive digital technology, and remote accommodations for people with disabilities. It brought to the forefront the prevailing disparities in access to education, healthcare, and employment, emphasizing the urgency of addressing those disparities to create equal opportunities and empowerment [7,8].

The digital transformation in higher education in Saudi Arabia, particularly in the context of Vision 2030, emphasizes inclusivity and accessibility. Despite advancements, challenges persist in ensuring full accessibility of university websites and digital health platforms for individuals with disabilities. Research indicates a wide range of challenges faced by web developers in enhancing web accessibility, including a lack of accessibility knowledge and negative attitudes towards disability issues [9,10]. Initiatives have been proposed to adapt web accessibility guidelines to the Arabic context, recognizing the cultural and linguistic differences that can impact accessibility [10]. Developing frameworks and checklists specific to local contexts aims to address the shortcomings in existing accessibility guidelines and enhance the overall accessibility of university websites in the country [11]. Moreover, research indicates that students with disabilities face barriers in accessing web-based information due to complex designs and incompatible assistive technologies [12]. Universities focusing on digital accessibility and providing technical support can enhance students’ browsing abilities, fostering academic and social competence [13]. The COVID-19 pandemic has heightened the need for digital inclusion, making accessible web designs crucial for supporting students with disabilities. Moreover, university websites can raise awareness about disability issues by showcasing inclusive policies and support services, inspiring the campus community to advocate for digital inclusion and foster a culture of social responsibility [14].

In this context, this study seeks to investigate the intersection of three crucial themes: (a) the rights and empowerment of people with disabilities, (b) the role of university websites, and (c) the lessons acquired from the COVID-19 pandemic. By exploring the experiences and responses of universities, specifically in the context of Saudi Arabia, we aim to shed light on the importance of accessible digital platforms during times of disaster. Based on this exploration, we offer recommendations for universities and institutions to better prepare for similar challenges in the future, thereby contributing to a more inclusive and sustainable society. The institutions selected for this exploration epitomize the diversity and dynamism of higher education across different countries: King Saud University (Saudi Arabia), Majmaah University (Saudi Arabia), the University of Oxford (United Kingdom), and Harvard University (United States). Geographic diversity was essential to capture a range of cultural contexts and regional approaches to disability empowerment, as cultural beliefs, values, practices, and regional factors significantly influence the implementation of disability support services [15]. By selecting universities from different regions, we aimed to explore how these contextual differences impact inclusivity and accessibility on university websites. Including comprehensive, research-focused, and emerging universities provided a holistic understanding of disability empowerment across various educational settings. Comprehensive universities typically serve diverse student populations, while research-focused institutions may have more resources and specialized programs. Emerging universities, like Majmaah, offer insights into the challenges and opportunities faced by newer institutions in implementing inclusive practices [14,16,17]. The selected universities are recognized for their global influence and leadership in higher education. This recognition was crucial as these institutions often globally set benchmarks and influence policies and practices in higher education. Their approaches to disability support can provide valuable lessons for other institutions seeking to enhance their inclusivity [14,16].

Our approach to university selection is characterized by a deliberate inclusion of institutions from diverse cultural and socioeconomic backgrounds, aimed at providing nuanced insights into the intricate landscape of disability empowerment. King Saud University and Majmaah University serve as representatives offering specific insights into the Saudi context, illuminating the unique challenges and strategies prevalent in the Middle Eastern region. Concurrently, the globally renowned University of Oxford and Harvard University contribute a vital international dimension to our study, enriching the comparative analysis. Majmaah University, positioned as an emerging institution, affords valuable perspectives into the hurdles associated with establishing inclusive practices. King Saud University, being a comprehensive institution, presents a distinctive viewpoint due to its expansive academic disciplines and programs. In contrast, the research-oriented nature of Oxford and Harvard deepens our understanding of disability empowerment strategies within such scholarly environments. By scrutinizing the content, initiatives, and narratives woven into the fabric of these websites, this study seeks to unravel how universities leverage their virtual spaces to foster inclusivity, advocate for the rights of individuals with disabilities, and contribute to broader societal change.

In this era of digital revolution and social progress, empowering individuals with disabilities remains a cornerstone of our common path to a more equal and just society. This study serves as a sworn statement to the critical role that university websites play in achieving this goal, especially in light of the significant impacts of the COVID-19 epidemic. By examining the context of digital health platforms for individuals with disabilities in Saudi Arabia, the U.K., and the U.S., we aim to provide a comprehensive comparison. The primary objective of this study is to investigate how university websites promote social responsibility and empower individuals with disabilities. Specifically, we aim to evaluate the accessibility services, inclusive policies, and academic accommodations provided by these websites. The research questions guiding this study are: (a) How do university websites foster inclusivity and advocate for disability rights? (b) How do these digital platforms contribute to societal change and support during disruptions such as the COVID-19 pandemic? This includes examining inclusive features, advocacy for disability rights, and societal impact facilitated by these websites, particularly in the post-COVID-19 pandemic context. Additionally, the study aims to identify any variations between universities in addressing these aspects.

## 2. Materials and Methods

### 2.1. Design

This study employed a qualitative design, specifically utilizing Thematic Analysis, as outlined by Braun and Clarke [18]. This approach is characterized by systematically identifying, analysing, and reporting patterns (themes) within the data. Thematic Analysis was chosen due to its flexibility and suitability for exploring how university websites promote social responsibility and empower individuals with disabilities. By examining the content, initiatives, and narratives presented on these websites, this research aims to uncover effective strategies for fostering inclusivity and advocating for disability rights. Although the technical aspects of website accessibility are crucial, incorporating criteria that specifically focus on the empowerment of individuals with disabilities is crucial for a comprehensive evaluation of university website. 

The conceptual framework for assessing the elements of accessibility on university websites was developed through a combination of data-driven insights and established principles from prior research. We conducted an extensive literature review on digital accessibility, inclusivity in higher education, and disability support services, drawing from guidelines by the World Wide Web Consortium (W3C) [19] and the Americans with Disabilities Act (ADA) [20], to gauge their inclusivity and provision of support for individuals with disabilities, as summarized in Table 1 [2,21,22,23,24]. From this review, we identified core elements essential for evaluating accessibility and inclusivity: accessibility services, inclusive policies, academic accommodations, empowerment initiatives, timely updates, community engagement, user-friendly access to support services, and success stories. These elements guided our systematic review and coding of the content from the official websites of the four universities. Through iterative discussions and refinement, we ensured the framework accurately reflected the diverse strategies employed by these universities. To validate the framework, a second researcher independently reviewed the coded data and themes, and an interrater reliability check was performed to enhance the validity and reliability of our findings. This comprehensive framework integrates empirical data with theoretical principles, offering a valuable tool for universities to enhance their digital platforms and support for individuals with disabilities.

In our study, the term “disabilities” encompasses a broad range of impairments, and our criteria are designed to provide a comprehensive evaluation applicable to various types of disabilities. The eight criteria for evaluating university websites focus on overarching aspects such as accessibility services, inclusive policies, academic accommodations, empowerment initiatives, timely updates, community engagement, user-friendly access to support services, and success stories. These criteria aim to capture the multifaceted nature of disability and ensure a thorough examination of university websites in promoting social responsibility and empowerment for individuals with diverse disabilities.

### 2.2. Data Sources

The primary data sources for this study were the official university websites of four distinct institutions: King Saud University (Saudi Arabia), Majmaah University (Saudi Arabia), the University of Oxford (United Kingdom), and Harvard University (United States). King Saud University, Majmaah University and the University of Oxford are public institutions, while Harvard University is a private, nonprofit institution, noting that in Saudi Arabia, public universities like King Saud University offer free education to Saudi citizens, while in the UK and the US, public universities provide various levels of financial aid and support to students with low economic resources. These universities are selected to represent diverse geographic regions and institutional characteristics. The selected universities, King Saud and Majmaah (representing Saudi Arabia), and Oxford and Harvard (globally recognized), were chosen for diversity in geographic regions and institutional characteristics. This diverse representation, including comprehensive (King Saud), research-focused (Oxford and Harvard), and emerging (Majmaah) institutions, facilitates a comprehensive exploration of disability empowerment across various contexts. The websites were navigated using a standardized protocol to ensure consistent data extraction. Data sources include website content, documents, and relevant multimedia materials available on the selected university websites.

### 2.3. Data Collection

The data collection process involved a thorough examination of the official university websites of the selected institutions. Information related to accessibility services, inclusive policies, academic accommodations, empowerment initiatives, timely updates and maintenance, community engagement, user-friendly access to support services, and success stories were extracted and analysed. Data collection and evaluation were anticipated to span six months, with information collection starting up in August 2023 and evaluation and write-up persevering with until February 2024.

### 2.4. Data Collection Procedure

1. A comprehensive review of each university’s official website was conducted to identify relevant sections related to disability support services, inclusive policies, academic accommodations, empowerment initiatives, timely updates and maintenance, community engagement, user-friendly access to support services, and success stories.
2. The content of these sections was systematically analysed, and relevant information was extracted, including textual content, images, videos, and downloadable resources.
3. The extracted data were organized into categories corresponding to the key areas of investigation.
4. All website content was systematically documented and organized. This involves recording text, images, and multimedia content.

### 2.5. Data Analysis

The extracted data underwent a combination of both qualitative content analysis and thematic analysis to identify recurring themes, patterns, and differences across the four universities. The analysis involved the following steps:

5. Data Coding: The extracted data were coded based on predefined categories, including accessibility services, inclusive policies, academic accommodations, empowerment initiatives, and success stories.
6. Theme Identification: Themes related to each category were identified through iterative coding and analysis. Commonalities and variations among the universities were documented.
7. Comparative Analysis: A comparative approach was used to compare the findings from each university. Patterns, trends, and differences in their approaches to promoting social responsibility and empowering individuals with disabilities were examined.
8. Rigor and Reliability: To ensure the robustness of our analysis, a second researcher conducted a comprehensive review of the coded data and themes. This collaborative effort aimed to enhance the validity of our findings. Additionally, an interrater reliability check was performed to minimize biases and strengthen the overall quality of the study. This process involved a thorough examination of the coding and theme identification, contributing to the credibility and trustworthiness of our data analysis.

### 2.6. Ethical Considerations

This study involved the analysis of publicly available information from university websites. No personal or sensitive data were collected, ensuring compliance with ethical standards. Ethical considerations included respecting the copyright and terms of use of website content.

## 3. Results

The analysis offered in the current study focuses on the four institutions’ inclusive practices and disability supporting measures, as detailed in the Appendix A Table A1. This comprehensive overview outlines each university’s performance across key criteria, providing a valuable reference for understanding their approaches to fostering inclusivity. The comparative analysis provides valuable insights into the diverse strategies employed by these universities to support individuals with disabilities and uphold principles of inclusivity. Considering inclusive policies and commitments, King Saud University demonstrates strong commitment through its Universal Access Program (UAP), offering bilingual information and various services. Majmaah University, while committed, can enhance communication specificity, especially in transportation. Conversely, Oxford and Harvard excel in detailed, accessible content, showcasing commitment through diverse communication channels.

The analysis of accessibility services and support across the four universities reveals distinct approaches. King Saud University emphasizes physical accessibility, assistive technologies, and individualized support through its UAP. Majmaah University demonstrates dedication with policies, private transportation, and e-training platforms, supported by positive testimonials. Oxford University’s DAS offers extensive guidelines and individualized support. Harvard University prioritizes digital accessibility and comprehensive employee support. Additionally, findings reveals that all universities are dedicated to accommodations, but detail varies on their websites. Moreover, the COVID-19 pandemic prompted significant adaptations in the accessibility policies and procedures of the universities studied. At King Saud University, the UAP played a crucial role in maintaining an accessible and supportive environment, addressing enrolment difficulties, legal rights, and administrative procedures, especially crucial during the pandemic. Majmaah University actively promoted inclusivity through policies, infrastructure improvements, and faculty/staff training. It enhanced e-training platforms and digital resources to support remote access to educational materials. The University of Oxford emphasized inclusivity through its DAS, supporting diverse needs, including mobility, sensory, mental health, and medical conditions. The pandemic led to an expansion of digital accessibility services and comprehensive guidelines on navigating online learning. Harvard University demonstrated its commitment through detailed information about accommodations and resources for creating inclusive content. Policies addressing physical accessibility, effective communication, and service animals were prioritized, ensuring that all students could fully participate in remote learning environments. These adaptations underscore the universities’ dedication to maintaining inclusivity and support for students with disabilities, highlighting the necessity for adaptable and resilient digital platforms during the global health crisis.

All four universities are dedicated to empowering students with disabilities through diverse initiatives, including training programs, collaborations, awareness campaigns, and resource provision. King Saud and Majmaah involve students in initiative development, recognizing diverse needs. University of Oxford excels with the Disability Law and Policy Project, emphasizing international collaboration and accessibility improvements. Harvard prioritizes digital accessibility and inclusive events.

Regarding website updates, King Saud has discrepancies, and Majmaah lacks explicit mention of update frequency. In contrast, the positive practices of Oxford and Harvard align with outlined strategies. Oxford’s community engagement and Harvard’s emphasis on up-to-date information demonstrate a proactive approach to website maintenance, supporting academic success and inclusion. The comparison underscores varying alignment with best practices, emphasizing consistent updates for web accessibility and accurate information dissemination.

Community engagement initiatives across the four universities involve students with disabilities in website development. King Saud University actively promotes knowledge transfer and community participation. Majmaah University emphasizes diversity through cross-departmental collaboration with government and non-governmental organizations. The University of Oxford and Harvard University exhibit active community engagement through campaigns, forums, and student alliances, incorporating diverse perspectives. Involving students with disabilities in website development, seen in King Saud University and Majmaah University, ensures accessibility.

King Saud University and Majmaah University prioritize clear information presentation and alternative channels for accessible support services, reflecting inclusivity. The University of Oxford encourages early registration with the DAS, ensuring timely support. Harvard University excels in organizing information and providing comprehensive insights into innovation projects. Challenges include King Saud University’s authentication requirement, potential accessibility issues with Majmaah University’s portal link, access problems with the University of Oxford’s “Access Student Self Service” feature, and restricted access to Harvard University’s LDC Portal.

While all universities present positive aspects in sharing success stories, there are variations in the level of detail and mediums used. King Saud University uses videos, emphasizing inclusivity with a success story of a visually impaired student. Majmaah University, while featuring success stories, can enhance impact with detailed testimonials. The University of Oxford employs a multifaceted approach, including profiles, interviews, and initiatives like the Let’s Get Disability on the List! campaign. Personal success stories highlight the importance of support services. Harvard University excels in comprehensive representation through websites, articles, and a dedicated book, emphasizing commitment through the Disability Justice Club’s advocacy efforts.

## 4. Discussion

Effective communication of inclusive policies and commitments is crucial for universities to ensure that students with disabilities are well-informed and supported. To enhance communication of inclusive policies, universities should ensure accessible website content, use clear language, employ multiple communication channels, and collaborate with disability services offices for effective dissemination of information to students with disabilities [25,26]. Our findings show that King Saud University demonstrates a strong commitment through its UAP, providing information in both English and Arabic and offering various services, which aligns with our recommendation for accessible website content. This was observed through the detailed bilingual information available on their website. Majmaah University, while showcasing its commitment through documents and testimonials, could enhance its communication by providing more specific details about services, especially those related to transportation for individuals with disabilities. This gap was noted in the limited details provided on their site compared to other institutions. On the other hand, the University of Oxford and Harvard University excel in providing detailed information through dedicated pages, aligning with the strategies of accessible website content, clear language, and multiple communication channels. Both universities have comprehensive sections detailing their inclusive policies and support services, which were evident in the extensive resources and support options listed.

To ensure accessibility services and support are accessible to all students with disabilities, universities should adopt various strategies. They should provide multiple modes of communication, ensuring physical accessibility, providing assistive technology, and collaborating with disability services offices [26]. Analysing four universities reveals King Saud and Majmaah’s commitment to physical and technological accessibility, while Oxford and Harvard emphasize personalized assistance and digital accessibility, reflecting the ongoing need for improvement in support services for students with disabilities. All universities display a commitment to accommodations, yet variations in website detail exist. Common adjustments, including prolonged exam time, note-taking assistance, accessible course materials, assistive technology, alternative testing formats, and flexible attendance policies, are addressed, which are consistent with the literature’s emphasis on personalised support [26,27,28,29]. For example, King Saud and Majmaah offer valuable support, with room for improvement in Majmaah’s specificity. Oxford and Harvard excel with comprehensive guidelines, showcasing an understanding of diverse needs. Support services beyond exams underscore a commitment to enhancing the overall learning experience. This underscores the significance of personalized and specialised care for students with disabilities.

To ensure effective empowerment initiatives for students with disabilities, universities should adopt collaborative development involving students [26], utilize multiple communication channels [28], collaborate with disability services offices [27], and provide training and support to staff and faculty [25]. These practices aim to tailor initiatives to individual needs, promote inclusivity, and enhance the overall success and inclusion of students with disabilities in higher education. The analysis of empowerment initiatives across the four universities reveals nuanced approaches to fostering inclusivity for students with disabilities. Each university is committed to creating an inclusive environment and supporting individuals with disabilities in academic pursuits. Practices in King Saud and Majmaah align with the existing literature, emphasizing collaborative development, communication channels, collaboration with disability services, and training. These contribute to a tailored and inclusive approach, promoting success and inclusion for students with disabilities in higher education. The findings underscore a universal commitment, offering insights for refining best practices and inspiring ongoing research into effective empowerment strategies in academia.

Timely updates and maintenance of university websites are crucial for students with disabilities as they ensure accessibility and usability, providing accurate information effectively. This is vital for academic activities such as accessing study materials and engaging in teaching and learning processes, especially in open distance learning institutions. Stakeholder involvement in website development is essential to ensure proper adherence to accessibility and usability standards, minimizing marginalization [30,31]. While all universities demonstrate efforts in maintaining updated information, there are areas for improvement, particularly in ensuring consistency across different sections, providing clearer information on the frequency of updates and addressing the currency of university-wide disability support services. These findings contribute valuable insights for enhancing the effectiveness of university websites in delivering timely and accurate information to users, including those with disabilities. The comparison highlights the varying degrees of alignment with best practices and the potential impact on students with disabilities, emphasizing the importance of consistent and timely updates across university websites.

Community engagement on university websites is crucial for benefiting students with disabilities. Involving them in website development ensures accessibility, usability, and timely information [30,31], reducing marginalization and promoting effective website use. Such engagement helps identify areas for improvement, fostering collaboration for an inclusive online environment [32,33]. Through such initiatives, universities can ensure equal opportunities for students with disabilities to access educational information and actively participate in academic activities [30,31,34]. Our findings show that the diverse community engagement strategies of these universities contribute to inclusivity. The impact on individuals with disabilities varies, requiring detailed initiatives for improvement. Coupled with a commitment to community engagement, this reduces marginalization, promoting effective website use. Emphasizing inclusivity and collaboration, observed in all universities, aligns with the literature. Community engagement significantly impacts students with disabilities, offering equal opportunities for information access and academic participation across contexts.

Ensuring user-friendly access to support services on university websites is crucial for fostering the equal participation of students with disabilities in both social and academic aspects of university life. Existing research underscores the profound impact of inadequate access and support services, which can lead to exclusion and dependency among students with disabilities [35,36]. Our evaluation of user-friendly access to support services across four universities reveals a spectrum of strengths and areas for improvement. These findings collectively underscore the critical importance of user-friendly access to support services for students with disabilities. The universities’ commitment to inclusivity, clarity, alternative options, and prompt issue resolution is crucial in fostering an accessible environment. Addressing the recognized challenges will further enhance the user experience, ensuring equitable access and guidance for all students, regardless of their competencies or disabilities.

Sharing success stories and testimonials on university websites is essential for supporting inclusivity, inspiring others, challenging stereotypes, showcasing capabilities, and highlighting effective disability support services [37]. Our evaluation reveals diverse strategies, using videos, articles, and initiatives demonstrating dedication to inclusivity and positive impact on students. In conclusion, universities must employ comprehensive strategies to communicate their commitment to supporting students with disabilities, contributing to a positive and inclusive campus environment.

The COVID-19 pandemic necessitated an examination of how higher education institutions responded to unprecedented challenges, particularly in the realm of disability support services and inclusive practices. One of the primary goals of this study was to identify and elucidate the lessons learned during this global crisis. Our analysis uncovered significant insights into the strategies employed by the selected universities to address the unique needs of students with disabilities amidst the pandemic. Universities rapidly adapted by offering remote assistive technology, virtual exam accommodations, and enhanced online resources. Consequently, the shift to remote learning modalities catalysed rapid adaptations in accessibility services and support mechanisms, highlighting the resilience and adaptability of higher education institutions in the face of adversity.

## 5. Conclusions

In the pursuit of understanding the complex interplay between the rights and empowerment of individuals with disabilities, the functionality of university websites, and the lessons learned from the challenges posed by the COVID-19 pandemic, this article conducts a thorough examination of four universities—King Saud University, Majmaah University, the University of Oxford, and Harvard University. Through the prism of Saudi Arabia’s context, we aim to shed light on the pivotal role of accessible digital platforms during times of crisis. By scrutinizing the responses and experiences of those institutions, we are searching for suggestions that can better equip universities for future difficulties contributing to the creation of a more inclusive and resilient society. The study underscores the critical role that university websites play in empowering individuals with disabilities, especially in light of the profound impacts of the COVID-19 pandemic. The comparative analysis reveals both effective practices and areas needing improvement across the universities, emphasizing a multifaceted approach encompassing accessibility, empowerment, timely updates, community engagement, user-friendly support services, and the promotion of success stories.

This study highlights several key contributions and practical implications. Firstly, enhancing accessibility and empowerment through digital platforms is crucial for providing students with disabilities with equal access to information, resources, and support services. Universities should prioritize digital accessibility to foster inclusivity. Secondly, regular updates and active community engagement are essential for maintaining the relevance and accessibility of university websites. Institutions must ensure their digital platforms are continuously updated and engage with the community to address the needs of students with disabilities. Lastly, sharing success stories of individuals with disabilities can inspire and motivate others, promoting a culture of inclusivity and empowerment within the academic community. The study’s findings have significant implications for policymakers in higher education. By adopting the recommended strategies, universities can create more inclusive environments that support the empowerment of students with disabilities. Policymakers should encourage institutions to prioritize digital accessibility and engage in continuous improvement of their support services.

## 6. Limitations and Future Research Directions

This study acknowledges several limitations. First, it focuses on a limited number of universities, which may not represent the broader spectrum of higher education institutions globally. Future research should include a more diverse sample of universities to enhance the representativeness and generalizability of the findings. Secondly, relying solely on publicly available information from university websites may not capture the full spectrum of disability support services. Future studies should incorporate primary data collection methods, such as surveys, interviews, and focus groups, to gain deeper insights. Additionally, the study is cross-sectional, capturing data at a single point in time, which does not account for changes in university policies over time. Longitudinal studies are needed to track the evolution of disability support services and the impact of digital platforms on inclusivity. Future research should also adopt a more comprehensive approach by examining how variations in accessibility policies and programs influence the representation, experiences, and outcomes for students with disabilities and other students. Investigating these dimensions will provide a holistic understanding of the effectiveness of these policies and programs. Specifically, future studies could explore how different policies and programs affect the representation of students with disabilities within the university community, their lived experiences, and their academic and personal outcomes. This approach will contribute significantly to developing evidence-based practices that support the inclusion and empowerment of students with disabilities. Future research should include universities with a high, medium, and low number of resources to identify best practices and areas for improvement. Anonymizing universities with fewer resources will ensure fair comparison while protecting their reputation. By addressing these limitations and incorporating diverse methods, the study aims to provide a comprehensive understanding of how universities can promote social responsibility and empower individuals with disabilities, particularly in the post-COVID-19 era, contributing to more inclusive educational policies and practices.

## Figures and Tables

**Table 1:** Criteria for evaluating university websites for social responsibility and empowerment of individuals with disabilities.

Criteria	Description
**Accessibility Services and Support**	- The resources and initiatives provided to ensure that individuals with disabilities have equal access to information, facilities, and opportunities.
2. **Inclusive Policies and Commitments**	- Formal guidelines and promises made by organizations, governments, or institutions to ensure equal participation and access for individuals with disabilities.
3. **Academic Accommodations**	- Adjustments and support provided to students with disabilities to ensure they have equal access to educational opportunities.
4. **Empowerment Initiatives**	- Programs and activities aimed at enhancing the confidence, independence, and decision-making abilities of individuals with disabilities.
5. **Timely Updates and Maintenance**	- Keeping information, facilities, and services current and in good working condition to ensure continued accessibility for individuals with disabilities.
6. **Community Engagement**	- The active participation and involvement of individuals with disabilities in the community.
7. **User-Friendly Access to Support Services**	- The ease of use and accessibility of resources and assistance available to individuals with disabilities.
8. **Success Stories and Testimonials**	- Accounts of individuals with disabilities who have achieved their goals, overcome challenges, or experienced positive outcomes with the support of accessibility services and accommodations.

## Data Availability

The data presented in this study are available within the manuscript.

## Appendix A

**Table A1:** Comparative analysis of disability support and inclusivity measures among the four universities.

Criteria	King Saud University (Saudi Arabia)	Majmaah University (Saudi Arabia)	University of Oxford (United Kingdom)	University of Harvard (United States)
**Accessibility Services and Support**	The Universal Access Program (UAP) focuses on creating an accessible environment with various services and facilities. Technical information and resources are available, showcasing a proactive approach in disseminating information about accessibility. The UAP conducts surveys touching upon enrolment difficulties, laws, and administrative procedures, indicating a comprehensive assessment of accessibility.	The Universal Access Program (UAP) focuses on creating an accessible environment with various services and facilities. Technical information and resources are available, showcasing a proactive approach in disseminating information about accessibility. The UAP conducts surveys touching upon enrolment difficulties, laws, and administrative procedures, indicating a comprehensive assessment of accessibility.	The Disability Advisory Service (DAS) offers one-on-one support, academic accommodations, and emphasizes eligibility criteria for receiving support. Provides accessible study resources, funding support, and resources for disability assessment, reflecting a comprehensive approach to accessibility. Emphasizes inclusivity by supporting diverse needs, including mobility, sensory, mental health, and medical conditions.	Harvard’s commitment is evident in providing detailed information about accommodations, accessibility initiatives, and resources for creating accessible digital content. Addresses physical accessibility with information about accessible features on campus, housing options, and transportation services. Demonstrates commitment in the workplace through accommodations, effective communication practices, and accessible workspaces. Provides resources for creating and delivering inclusive content, reflecting a commitment to innovation in enhancing accessibility.
**Inclusive Policies and Commitments**	Inclusive policies are evident through the UAP’s role in creating an accessible and supportive environment. The university’s survey includes aspects of inclusivity, enrolment difficulties, legal rights, and administrative procedures.	Active engagement in promoting inclusivity within its academic community, demonstrated in policies, infrastructure improvements, and faculty/staff training. Policies and regulations protect the rights of people with disabilities, fostering inclusivity in admissions, academic support services, and faculty/staff training.	Emphasizes inclusivity and equal access through eligibility criteria, resources, and support services provided by the DAS. Supports diverse needs, including mobility, sensory, mental health, and medical conditions, reflecting a commitment to inclusive policies.	Demonstrates commitment through detailed information about accommodations, eligibility criteria, and resources for creating inclusive content. Addresses physical accessibility, effective communication, and policies for service animals, reflecting a commitment to inclusivity in various aspects.
**Academic Accommodations**	Provides information on requesting accommodations, emphasizing support for students with disabilities. The UAP facilitates various academic accommodations, such as extended exam time, accessible course materials, and individualized support. Highlights multiple accommodations, including note-taking support, sign language interpretation, and accessible transportation. Information on academic accommodations and support services is available in Arabic. Discusses efforts to provide academic accommodations, such as accessible learning resources, training for faculty members, and assistive technology.	The document on Supporting Students with Special Needs emphasizes allocating private transportation and a driver as academic accommodations. Demonstrates a focus on evaluating students based on their abilities and providing a suitable environment for students with special needs. Training for faculty and staff aims to enhance awareness of the needs of individuals with disabilities, promoting a more inclusive learning experience. While the platform and visit to “Wusul” are informative, they do not explicitly detail the university’s approach to academic accommodations for people with disabilities.	Offers detailed guidelines for various disabilities and highlights the DAS’s role in providing advice, arranging academic adjustments, and supporting students throughout their studies. Discusses academic accommodations, including adjustments for exams, coursework, alternative formats, and note-taking support. Focuses on providing academic accommodations and adjustments tailored to individual needs, with a strong emphasis on exam adjustments and study techniques.	Provides comprehensive guidelines for various types of disabilities and focuses on academic accommodations, including exam adjustments, accessible course materials, and note-taking support. Offers contact details for inquiries, emphasizing digital accessibility. Discusses physical accessibility on campus, event planning guidance, and workplace accommodations rather than academic accommodations. Highlights the provision of course materials in alternative formats.
**Empowerment Initiatives**	Through its UAP, the university is committed to empowering students with disabilities. The UAP conducts various initiatives, including training programs, workshops, and conferences, to enhance the skills and awareness of students. The program’s mission, outlined in Arabic, emphasizes creating a supportive environment, adhering to international standards, and overcoming barriers to ensure the full participation of students with disabilities.	Testimonials reflect the university’s success in providing resources to overcome challenges, while documents and initiatives like “Achieve Your Ambition” demonstrate a commitment to creating an inclusive environment. Collaborations with organizations, awareness campaigns, and events contribute to changing societal attitudes. The e-training platform and disability benefits further align with empowerment goals, providing educational content and financial support to students with disabilities.	The Oxford University Disability Law and Policy Project at the University of Oxford stands out as a comprehensive initiative. It not only showcases a commitment to improving accessibility but also focuses on diverse perspectives, inclusivity in research and teaching, and international collaboration. This project positions the university as a model for promoting disability empowerment within academic institutions.	Harvard University’s emphasis on creating accessible digital content, planning inclusive events, and providing resources for inclusive content creation aligns with empowerment principles. The university’s focus on innovation and pilot projects further underscores its commitment to empowering individuals with disabilities.
**Timely Updates and Maintenance**	The evaluation of King Saud University’s website indicates variations in the last update dates across different pages. While some sections display recent updates, others show discrepancies, suggesting an area for improvement in maintaining consistency in updates across the website. This inconsistency could potentially impact users seeking the latest information.	The information provided for Majmaah University lacks explicit details on the frequency or regularity of updates, raising the importance of ensuring that information related to support services is regularly updated for accuracy. Some events are dated back to 2020 and 2021, indicating a need for more frequent updates. The e-training platform, disability benefits policy, and accessibility services lack clarity on the frequency of updates or regular reviews, highlighting areas for improvement.	The University of Oxford actively engages with the community, exemplified by initiatives like the Let’s Get Disability on the List! campaign and the Knowledge Exchange Forum. While these efforts demonstrate commitment to diversity and inclusion, the information does not explicitly address the frequency of updates on university-wide disability support services, indicating a potential area for improvement.	Harvard University’s content appears up to date, featuring various initiatives, clubs, and services. The “Innovation Pilot Projects” page showcases a commitment to ongoing improvements with projects presented at varying timelines. The inclusion of specific information for various Harvard schools suggests an effort to provide up-to-date information for students seeking accommodations. The article on “Addressing Disability” discusses recent events, affirming the university’s commitment to staying current with disability-focused initiatives.
**Community Engagement**	King Saud University actively engages in collaborations with external entities, signing cooperation agreements with a publishing and translation company and the Family Affairs Council. These initiatives demonstrate the university’s commitment to knowledge sharing and fostering community collaborations. The university seeks insights into the experiences of students with disabilities through surveys, evaluating engagement levels and addressing challenges within the university community.	Majmaah University aims to create a comprehensive university community by fostering inclusivity and promoting awareness about disabilities. Collaboration with various university departments and faculties is encouraged to support students with special needs. The university’s partnerships with governmental and non-governmental organizations, along with hosting forums and events focused on women with disabilities, showcase an active involvement in the broader community. However, specific impacts on individuals with disabilities from collaborations, such as with the Sheikh Ibrahim Al-Sultan Charitable Foundation, need further detailing.	The University of Oxford actively engages with the community, focusing on the inclusion and support of individuals with disabilities. Initiatives like the “Let’s Get Disability on the List!” campaign, the Knowledge Exchange Forum, and the Disability Law and Policy Project demonstrate the university’s commitment to diverse perspectives and community involvement.	Harvard University showcases community engagement through student groups like the Harvard College Disability Alliance and the Harvard Undergraduate Disability Justice Club. Projects, including building a participant pool for accessibility testing, highlight engagement and collaboration within the Harvard community. The mention of Local Disability Coordinators (LDCs) and the interactive process for accommodation requests further implies community engagement and collaboration.
**User-Friendly Access to Support Services**	Access to support services requires authentication through a university email, potentially creating a barrier for users without one. The ‘Consultation phone (WhatsApp)’ service offers an alternative and user-friendly option for accessible consultations. Site surveys assess the availability, effectiveness, and importance of support services, providing user-driven perspectives for improvement.	Information about support services for students with special needs is presented clearly on the website. A dedicated portal exists for students with special needs, but the link is not mentioned, potentially affecting accessibility. The YouTube channel may serve as a user-friendly access point, but website ease of use is not explicitly addressed.	The university encourages early registration with the Disability Advisory Service, offering accessible support for award-bearing and non-award-bearing courses. The “Access Student Self Service” feature has an access issue, promptly communicated with clear contact information for issue resolution.	Well-organized information about disability support services is available across different schools and departments. Clear contact information is provided for disability services, including the Accessible Education Office. Comprehensive details about innovation pilot projects for digital access. The “Student Accommodation Coordinators” page offers information on accommodation and clear contact details. The “Addressing Disability” article recognizes key figures in disability28 advocacy and emphasizes ongoing communication channels.
**Success Stories and Testimonials**	The university showcases success stories through videos, including an overview of services for individuals with disabilities, achievements of students with disabilities, and an initiative for visually impaired students and staff. A success story of a visually impaired student and his achievements is featured, contributing to a positive narrative about the university’s commitment to inclusivity.	The webpage provides glimpses of success stories, with students sharing their experiences and ambitions. However, more detailed testimonials could be highlighted to inspire and motivate others. Videos, including an overview of services and testimonials from graduates with special needs, serve as success stories, showcasing the positive impact of the university on the academic and personal lives of students with special needs.	The university presents success stories through profiles and interviews of students who have overcome challenges and achieved success. Initiatives like the Let’s Get Disability on the List! campaign and the Knowledge Exchange Forum aim to incorporate disability perspectives into academic outputs and research. Personal success stories include a student who overcame significant challenges after a major car accident, emphasizing the importance of support services and individualized assistance in their journey to pursuing a doctorate.	Success stories and testimonials for disabled students at Harvard University are featured on various websites, including articles on student- and alumni-led accessibility activism. The book “How Did You Get Here? Students With Disabilities and Their Journeys to Harvard” provides interviews with Harvard students with disabilities, offering valuable insights into their experiences and perseverance. The Harvard Undergraduate Disability Justice Club advocates for increased campus visibility, improved building accessibility, and more inclusive policies, contributing to the university’s commitment to supporting disabled students.
